# Occult blood in feces is associated with a poor functional outcome of ischemic stroke patients receiving intravenous thrombolysis

**DOI:** 10.3389/fneur.2025.1533933

**Published:** 2025-03-31

**Authors:** Min Peng, Xiaoyun Sun, Xiangling Yuan, Chenjuan Tao, Gelin Xu

**Affiliations:** ^1^Department of Neurology, Institute of Translational Medicine, The First Affiliated Hospital of Shenzhen University, Shenzhen Second People's Hospital, Shenzhen, China; ^2^Department of Neurology, The Affiliated Hospital of Hangzhou Normal University, Hangzhou, China; ^3^Department of Anesthesiology, Nanjing Women and Children’s Healthcare Hospital, Nanjing, Jiangsu, China; ^4^Guangxi University of Chinese Medicine, Nanning, China

**Keywords:** fecal immunochemical test, acute ischemic stroke, outcome, the modified Rankin Scale, intravenous thrombolysis

## Abstract

**Background:**

Although fecal occult hemoglobin is commonly valued as a screening tool for colorectal cancer, few studies have examined the clinical significance of fecal immunochemical testing (FIT) in other diseases. This study aimed to explore the association between occult blood in feces and functional outcomes of acute ischemic stroke (AIS) patients who received intravenous thrombolysis treatment.

**Methods:**

Patients diagnosed with acute ischemic stroke and received thrombolytic therapy were recruited from the neurology department of the Affiliated Hospital of Hangzhou Normal University. FIT was conducted for patients during hospitalization. Functional outcome was assessed by the modified Rankin Scale (mRS). A favorable outcome was defined as mRS 0–2 and a poor outcome as mRS 3–6.

**Results:**

A total of 214 patients were included for analysis. The proportion of FIT-positive patients was higher in the poor outcome group than in the favorable group (12.3% vs. 45.6%, *p* < 0.001). Logistic regression models showed that FIT-positive patients had an increased risk of a poor outcome (OR: 4.188, 95% CI: 1.424–11.51, *p* = 0.005) after adjusting for possible variables. Moreover, in addition to gastrointestinal bleeding, NIHSS score at baseline (OR: 1.092, 95% CI: 1.013–1.176, *p* = 0.021) and white blood cell level (OR: 1.215, 95% CI: 1.018–1.448, *p* = 0.031) were also the independent risk factors for positive FIT after thrombolytic therapy in AIS.

**Conclusion:**

Positive FIT was related to the poor outcomes in AIS patients who received thrombolytic therapy. High NIHSS scores at baseline and high white blood cell levels were the risks of FIT.

## 1 Introduction

Stroke is a common disease that causes high mortality and high disability, and the number of strokes and stroke-related deaths is rising significantly worldwide (1). In 2021, stroke contributed to 7.3 million deaths, making it the third leading cause of death worldwide (2). Depending on the nature of the underlying lesion, strokes can be classified as ischemic or hemorrhagic. There were 7.8 million new ischemic strokes globally in 2021 and was the prevalent stroke subtype (2). Therefore, it is necessary to understand, predict, and improve functional outcomes in patients with acute ischemic stroke (AIS).

Fecal immunochemical test (FIT) is mainly used for the diagnosis of gastrointestinal bleeding (GIB) and colorectal tumors, and there are two commonly used methods, i.e., immunoassay and chemical assay (3). Many countries have implemented extensive CRC screening initiatives that use FITs. The U.S. Preventive Services Task Force recommends annual FIT screening in average-risk individuals to reduce the risk of colorectal cancer (4). FIT can help physicians be informed of a patient’s occult intestinal bleeding, which can guide treatment.

Intravenous thrombolysis (IVT) with alteplase within 4.5 h from the onset according to the worldwide stroke guidelines is one of the most important treatments in the management of AIS (5). However, there is a risk of subcutaneous petechiae, gingival bleeding, and intracranial hemorrhage (ICH) occurring with either urokinase or alteplase use.

Stroke and CR share several risk factors, such as advanced age, smoking, and dietary habits, indicating that they may have the same pathologic mechanisms. Previous evidence on occult blood in feces and prognosis after IVT in ischemic stroke is scarce. Therefore, the aim of this study was to investigate the correlation on positive fecal occult blood and its prognosis in AIS received IVT.

## 2 Methods

### 2.1 Study population

Patients were enrolled from January 2021 to May 2024 from the neurology department of the Affiliated Hospital of Hangzhou Normal University. The inclusion criteria were as follows: patients with (1) age ≥ 18 years; (2) diagnosed with AIS through head CT or head magnetic resonance imaging (MRI); and (3) thrombolytic therapy after admission. The exclusion criteria were patients with pre-existing gastrointestinal conditions or recent use of anticoagulants. The study protocol was approved by the Ethics Committee of the Affiliated Hospital of Hangzhou Normal University. Patient informed consent was not required because this study was retrospective.

### 2.2 FIT measurements

Patients’ stool samples were typically collected within a week after thrombolysis, and the collected samples of each individual were sent to the Laboratory Department for measurement within 2 h. FITs were processed using a 1-day quantitative sampling method; 3 g of feces was collected with disposable sterile cotton swabs and stored in sterile containers. Fecal samples were assayed for blood in feces following an established protocol using an automatic biochemistry analyzer HALO-F280 (Suzhou Halo Biological Technology Co., Suzhou, China). One-Step Fecal Occult Blood (FOB) kits (Suzhou Halo Biological Technology Co., Suzhou, China) were used for the quantitative method. The results were divided into negative and positive according to the analyzer.

### 2.3 Clinical outcome assessments

The Baseline National Institutes of Health Stroke Scale (NIHSS) scores were assessed by a specialty-trained and experienced neurologist prior to treatment for stroke severity. All neurologists have at least 3 years of clinical experience. All patients were given standardized neurological treatment. Patients who were eligible for recanalization treatment would receive thrombolysis or thrombectomy. Thrombolytic therapy was defined as the intravenous administration of thrombolytic agents such as recombinant tissue-type plasminogen activator (rtPA). Neurological functional outcome was assessed by the modified Rankin Scale (mRS) (range: 0 to 6) at 90 days post-stroke. mRS assessment was performed by outpatient visit or by telephone by a trained neurologist. A 90-day mRS score of 0–2 is considered a favorable prognosis, whereas 3–6 is considered a poor prognosis.

### 2.4 Covariates

Covariates such as age, sex, vascular risk factors [smoking status, diabetes mellitus, hypertension, atrial fibrillation, coronary heart disease (CHD) history, and stroke history], time to intravenous thrombolysis (IVT) from the onset, random blood glucose (RBG) on admission, systolic blood pressure (SBP) on admission, diastolic blood pressure (DBP) on admission, rtPA dose, medication pre-admission (anticoagulation/antiplatelet agent, statin, hypoglycemic agent, and antihypertensive agent), NHISS score at three time points (baseline, 1 h after IVT, and 7 days after IVT), mechanical embolectomy, GIB, and intracranial hemorrhage were included. Anyone who reported smoking cigarettes prior to or during admission was classified as a smoker. A fasting blood glucose level of ≥126 mg/dL, a known history of diabetes mellitus, and the use of hypoglycemic medications to regulate blood sugar were all considered indicators of diabetes mellitus (6). A history of hypertension, a systolic blood pressure ≥ 140 mm Hg, a diastolic blood pressure ≥ 90 mm Hg, and the use of antihypertensive medications were all considered indicators of hypertension (7). Atrial fibrillation was defined by ECG detection of atrial fibrillation or self-report (8). Self-report of myocardial infarction, percutaneous coronary intervention, coronary artery bypass graft surgery, or ECG evidence of myocardial infarction were used to characterize coronary heart disease (9). Stroke history was defined as patients who had a history of stroke. GIB was defined as a hospitalization record of the upper or lower GIB, regardless of the cause of bleeding (10). Intracranial hemorrhagic transformation was defined as parenchymal hemorrhage of the infarct tissue (11).

Laboratory parameters were measured in fasting venous blood in the early morning of the second day of admission. White blood cell (WBC), neutrophil-to-lymphocyte ratio (NLR), C-reactive protein (CRP), albumin, homocysteine (Hcy), uric acid, hemoglobin Alc (HbA1c), fasting blood glucose (FBG), serum total cholesterol, serum triglyceride, high-density lipoprotein cholesterol (HDL-C), and low-density lipoprotein cholesterol (LDL-C) were collected.

### 2.5 Statistical analysis

Data were shown as numbers (percentage) or mean ± standard deviation (m ± SD). The differences in continuous and categorical variables were compared using Student’s *t*-test and the chi-square test, respectively.

Logistic regression analysis was used to evaluate the association of FIT and outcome. Age, sex, and variables with *p*-values <0.1 in the univariate analysis were included in the logistic regression. For model 1, we adjusted for age and sex. In model 2, the adjusted variables were age, sex, smoking ever, atrial fibrillation, RBG on admission, rtPA dose, NIHSS score at baseline, WBC, NLR, CRP, uric acid, mechanical embolectomy, GIB, and intracranial hemorrhage. To explore the risk factors for positive FIT, age and sex as well as *p*-values <0.1 in the univariate analysis such as atrial fibrillation, stroke history, DBP on admission, anticoagulation/antiplatelet agent, antihypertensive agent, NIHSS score at baseline, WBC, NLR, CRP, triglyceride, HDL-C, GIB, and intracranial hemorrhage were screened into the models. All statistical analyses were performed using IBM SPSS Statistics 22 (PASW 22, SPSS Inc., IBM, Armonk, NY, United States).

## 3 Results

### 3.1 Characteristics of the study cohort grouped on mRS

In total, 214 AIS patients who received IVT with a mean (SD) age of 63.1 (14.8) years were included for analysis, and 72.9% of them were male. A total of 49 (22.9%) had a positive FIT, and 77.1% of patients had a favorable outcome whereas 22.9% of patients had a poor outcome.

As shown in Table 1, compared with the favorable outcome patients, patients with a poor outcome were more likely to have a lower ratio of smoking history (30.9% vs. 48.6%, *p* = 0.015), but a higher ratio of atrial fibrillation (32.4% vs. 13.0%, *p* = 0.001), mechanical embolectomy (14.7% vs. 2.7%, *p* = 0.002), GIB (19.1% vs. 3.4%, *p* < 0.001), and intracranial hemorrhage (0 vs. 13.2%, *p* < 0.001). In addition, the poor outcome group had more severe stroke symptoms on admission, 1 h, and 7 days after IVT than the favorable outcome group (all *p* < 0.001). In addition, patients with a favorable outcome group had a lower concentration of WBC (*p* < 0.001), CRP (*p* = 0.045), and FPG (*p* < 0.001) and a higher level of uric acids (*p* = 0.036) than a poor outcome group. The positive FIT rate of the poor outcome group was also higher than the favorable outcome group.

**Table 1:** Baseline characteristics according to clinical outcome (*N* = 214).

Characteristics	Favorable outcome (mRS 0–2)	Poor outcome (mRS 3–6)	*P-*value
No.	146 (77.1)	68 (22.9)	
Age (y)	62.0 ± 14.9	65.5 ± 14.4	0.113
Men	112 (76.7)	44 (64.7)	0.066
Vascular risk factors			
Smoking ever	71 (48.6)	21 (30.9)	0.015
Diabetes mellitus	28 (19.2)	12 (17.6)	0.789
Hypertension	90 (61.6)	44 (64.7)	0.666
Atrial fibrillation	19 (13.0)	22 (32.4)	0.001
CHD history	13 (8.9)	9 (13.9)	0.331
Stroke history	19 (13.0)	9 (13.2)	0.964
Time to IVT from onset (min)	163 ± 624	166 ± 57.4	0.710
RBG on admission (mmol/L)	7.52 ± 2.43	8.24 ± 2.86	0.069
SBP on admission (mmHg)	153 ± 23.8	155 ± 24.4	0.496
DBP on admission (mmHg)	89.1 ± 15.4	85.4 ± 17.3	0.117
rtPA dose (mg)	63.3 ± 13.2	56.6 ± 12.5	0.008
Medication pre-admission			
Anticoagulation/antiplatelet agent	19 (13.0)	13 (19.1)	0.244
Statin	10 (6.8)	5 (7.4)	0.893
Hypoglycemic agent	13 (8.9)	8 (11.8)	0.513
Antihypertensive agent	70 (47.9)	32 (47.1)	0.904
NIHSS score			
At baseline	5.76 ± 4.61	14.3 ± 6.74	<0.001
1 h after IVT	4.03 ± 4.16	13.8 ± 7.02	<0.001
7 days after IVT	2.06 ± 2.39	13.4 ± 7.53	<0.001
Laboratory parameters			
WBC (×10^9^/L)	7.62 ± 2.23	9.89 ± 3.39	<0.001
NLR	4.32 ± 2.86	9.78 ± 10.6	<0.001
CRP (mg/L)	5.88 ± 10.9	9.96 ± 1.47	0.045
Albumin	37.8 ± 3.96	37.5 ± 4.52	0.647
Hcy (μmol/L)	14.8 ± 11.2	16.4 ± 14.7	0.447
Uric acid (mmol/L)	357 ± 108	324 ± 100	0.036
HbA1c (mmol/L)	6.11 ± 1.07	6.19 ± 1.15	0.597
FPG (mmol/L)	6.06 ± 2.42	7.78 ± 3.23	<0.001
Total cholesterol (mmol/L)	4.33 ± 0.90	4.42 ± 1.16	0.573
Triglyceride (mmol/L)	1.50 ± 0.94	1.30 ± 0.67	0.118
HDL-C (mmol/L)	1.11 ± 0.40	1.16 ± 0.28	0.435
LDL-C (mmol/L)	2.59 ± 0.68	2.66 ± 0.83	0.565
Mechanical embolectomy	4 (2.7)	10 (14.7)	0.002
Gastrointestinal bleeding	5 (3.4)	13 (19.1)	<0.001
Intracranial hemorrhage	0 (0)	9 (13.2)	<0.001
FIT (+)	18 (12.3)	31 (45.6)	<0.001

CHD, coronary heart disease; IVT, intravenous thrombolysis; RBG, random blood glucose; SBP, systolic blood pressure; DBP, diastolic blood pressure; rtPA, recombinant tissue-type plasminogen activator; NIHSS, National Institutes of Health Stroke Scale; WBC, white blood cell; NLR, neutrophil-to -lymphocyte ratio; CRP, C-reactive protein; Hcy, homocysteine; HbA1c, hemoglobin Alc; FBG, fasting blood glucose; HDL-C, high-density lipoprotein cholesterol; LDL-C, low-density lipoprotein cholesterol; FIT, fecal immunochemical test.

### 3.2 Logistic regression analysis between positive FIT and outcome

The participants with positive FIT had more significant mortality risks of a poor outcome (OR: 5.958, 95% CI: 2.999–11.84) in the crude model. The association was still positive after adjusted age and sex (OR: 5.872, 95% CI: 2.933–11.76) in model 1. After adjusting for all possible confounders, patients with positive FIT had greater risks of a poor outcome (OR: 4.188, 95% CI: 1.424–11.51) (Table 2).

**Table 2:** Logistic regression analysis for positive FIT predicting a poor outcome.

Poor outcome (mRS 3–6)	OR (95% CI)	*P-*value
Crude model	5.958 (2.999–11.84)	<0.001
Model 1^a^	5.872 (2.933–11.76)	<0.001
Model 2^b^	4.188 (1.424–11.51)	0.005

^aModel 1 adjusted for age and sex.^

^bModel 2 adjusted for age, sex, smoking ever, atrial fibrillation, RBG on admission, rtPA dose, NIHSS score at baseline, WBC, NLR, CRP, uric acid, mechanical embolectomy, gastrointestinal bleeding, and intracranial hemorrhage.^

RBG, random blood glucose; rtPA, recombinant tissue-type plasminogen activator; NIHSS, National Institutes of Health Stroke Scale; WBC, white blood cell; NLR, neutrophil-to-lymphocyte ratio; CRP, C-reactive protein.

### 3.3 Characteristics of the study cohort grouped on FIT results

The FIT-positive patients had a higher proportion of atrial fibrillation (*p* = 0.006), higher diastolic blood pressure levels (*p* = 0.041), and higher NIHSS scores at three time points (*p* < 0.041) than the FIT-negative group. As for laboratory parameters, the FIT-positive group had a higher level of WBC (*p* = 0.001), NLR (*p* = 0.017), and CRP (*p* = 0.006) and a lower level of triglyceride (*p* = 0.009). In addition, the rate of GIB (*p* < 0.001) and intracranial hemorrhage (*p* = 0.031) was greater in the FIT-positive group than in the FIT-negative group (Table 3).

**Table 3:** Baseline data among study population according to FIT results (*N* = 214).

Characteristics	FIT (−)	FIT (+)	*P-*value
No.	165 (77.1)	49 (22.9)	
Age (y)	62.4 ± 14.8	65.5 ± 14.8	0.196
Men	122 (73.9)	34 (69.4)	0.529
Vascular risk factors			
Smoking ever	72 (43.6)	20 (40.8)	0.726
Diabetes mellitus	33 (20.0)	7 (14.3)	0.368
Hypertension	99 (60.0)	35 (71.4)	0.147
Atrial fibrillation	25 (15.2)	16 (32.7)	0.006
CHD history	15 (9.1)	7 (14.3)	0.293
Stroke history	18 (10.9)	10 (20.4)	0.083
Time to IVT from onset (min)	168 ± 62.9	151 ± 57.8	0.100
RBG (mmol/L)	7.68 ± 2.61	7.94 ± 2.48	0.564
SBP on admission (mmHg)	153 ± 23.9	154 ± 24.5	0.900
DBP on admission (mmHg)	89.2 ± 16.4	83.8 ± 14.3	0.041
rtPA dose (mg)	61.7 ± 17.7	59.5 ± 14.0	0.437
Medication pre-admission			
Anticoagulation/antiplatelet agent	21 (12.7)	11 (22.4)	0.094
Statin	10 (6.1)	5 (10.2)	0.342
Hypoglycemic agent	16 (9.7)	5 (10.2)	1.000
Antihypertensive agent	73 (44.2)	29 (59.2)	0.066
NIHSS score			
Baseline	7.25 ± 5.72	12.7 ± 8.13	<0.001
1 h after IVT	5.83 ± 5.74	11.5 ± 8.68	<0.001
7 days after IVT	4.24 ± 5.67	9.98 ± 9.00	<0.001
Laboratory parameters			
WBC (×10^9^/L)	7.88 ± 2.42	9.92 ± 3.61	0.001
NLR	5.09 ± 4.22	9.34 ± 11.8	0.017
CRP (mg/L)	5.33 ± 8.81	13.4 ± 19.2	0.006
Albumin	37.9 ± 4.01	37.0 ± 4.52	0.193
Hcy (μmol/L)	15.1 ± 12.6	16.1 ± 11.8	0.602
Uric acid (mmol/L)	346 ± 104	350 ± 117	0.829
HbA1c (mmol/L)	6.14 ± 1.16	6.12 ± 0.86	0.908
FPG (mmol/L)	6.40 ± 2.57	7.28 ± 3.44	0.107
Total cholesterol (mmol/L)	4.34 ± 0.99	4.42 ± 1.00	0.650
Triglyceride (mmol/L)	1.49 ± 0.96	1.23 ± 0.46	0.009
HDL-C (mmol/L)	1.10 ± 0.27	1.22 ± 0.61	0.064
LDL-C (mmol/L)	2.61 ± 0.71	2.64 ± 0.78	0.809
Mechanical embolectomy	11 (6.7)	3 (6.1)	1.000
Gastrointestinal bleeding	1 (0.6)	17 (34.7)	<0.001
Intracranial hemorrhage	4 (2.4)	5 (10.2)	0.031
Clinical outcomes at 90 days			
Unfavorable outcomes (mRS 3–6)	42 (25.5)	34 (69.4)	<0.001
Mortality (mRS 6)	10 (6.1)	7 (14.3)	0.064

FIT, fecal immunochemical test; CHD, coronary heart disease; IVT, intravenous thrombolysis; RBG, random blood glucose; SBP, systolic blood pressure; DBP, diastolic blood pressure; rtPA, recombinant tissue-type plasminogen activator; NIHSS, National Institutes of Health Stroke Scale; WBC, white blood cell; NLR, neutrophil-to-lymphocyte ratio; CRP, C-reactive protein; Hcy, homocysteine; HbA1c, hemoglobin Alc; FBG, fasting blood glucose; HDL-C, high-density lipoprotein cholesterol; LDL-C, low-density lipoprotein cholesterol.

### 3.4 Logistic regression to explore the risk factors for positive FIT

We explored the risk factors for a positive FIT and found that, in addition to GIB, high NIHSS scores at baseline (OR: 1.092, 95% CI: 1.013–1.176) and elevated WBC levels (OR: 1.215, 95% CI: 1.018–1.448) were associated with positive FIT (Table 4).

**Table 4:** Logistic regression to explore the risk factors for positive FIT.

Variables	OR (95% CI)	*P-*value
Age	0.985 (0.952–1.019)	0.379
Men	1.323 (0.487–3.595)	0.583
Atrial fibrillation	1.889 (0.657–5.434)	0.238
Stroke history	1.935 (0.556–6.737)	0.300
DBP on admission	0.982 (0.957–1.009)	0.188
Anticoagulation/antiplatelet agent	1.300 (0.391–4.325)	0.669
Antihypertensive agent	1.832 (0.760–4.416)	0.177
NIHSS score at baseline	1.092 (1.013–1.176)	0.021
WBC	1.215 (1.018–1.448)	0.031
NLR	0.935 (0.846–1.032)	0.181
CRP	1.003 (0.964–1.043)	0.892
Triglyceride	0.836 (0.472–1.481)	0.539
HDL-C	1.768 (0.676–4.624)	0.246
Gastrointestinal bleeding	65.86 (6.205–699.0)	0.001
Intracranial hemorrhage	0.678 (0.060–7.594)	0.752

NIHSS, National Institutes of Health Stroke Scale; WBC, white blood cell; NLR, neutrophil-to-lymphocyte ratio; CRP, C-reactive protein; HDL-C, high-density lipoprotein cholesterol.

## 4 Discussion

To our knowledge, this is the first study to reveal that FIT was an independent factor of neurological functional outcome in the patients with AIS who received IVT. In addition, high NIHSS scores at baseline and high WBC levels were the risks for positive FIT after IVT. Positive-FIT patients after IVT had 4-fold odds of gaining a poor outcome than the negative-FIT patients, and patients with high NIHSS scores and high WBC levels were approximately 1.1 and 1.2 times more likely to have a positive FIT, respectively.

Few studies have been done on the association between FIT and ischemic stroke, but there was considerable research on GIB and ischemic stroke (12, 13). Only one previous literature has demonstrated that the positive FIT results were associated with an increased risk of ischemic stroke, myocardial infarction, and all-cause mortality (14). GIB is a well-known complication that can occur during the acute phase of AIS. The prevalence of GIB in ischemic stroke ranged from 1.5 to 7.8% (13, 15). Stroke patients with GIB may also have elevated WBC counts and elevated FBG levels (16). The explanation may be that pro-inflammatory cytokine release leads to mucosal ulceration. GIB in AIS patients portended a worse prognosis. GIB can lead to neurological deterioration, prolonged hospitalization, poor functional outcomes, and increased disability at 3 months (16, 17). The findings are similar to ours. Remarkably, patients with AIS who had GIB exhibit higher long-term mortality rates (18). Siddiqui et al. (19) suggested that patients with GIB could be risk stratified by gastrointestinal endoscopy (GIE), but AIS was considered a relative contraindication to GIE.

The underlying mechanisms between occult blood in feces and poor outcomes are not completely understood, which may be explained by systemic inflammation. Increased levels of inflammatory factors can lead to mucosal damage. Therefore, a positive FIT result can be interpreted as systemic intestinal inflammation with occult bleeding. Moreover, ischemic stroke, myocardial infarction, and their outcomes may be caused by a similar possible mechanism of the systemic inflammatory sequence. The initiating factor in the formation of atherosclerotic plaques is inflammation. Thus, positive FIT results as a marker of inflammatory response can be associated with a poor prognosis. In addition, the gastrointestinal state after stroke is closely related to the gut microbiota. The gut microbiota regulates neuroinflammation through the bidirectional microbiota–gut–brain axis, potentially affecting stroke outcomes. Several studies have demonstrated that stroke induces alterations in the composition of gut microbiota, early loss of the host intestinal barrier, increased bacterial translocation to the host, and changes in the host–microbiota metagenomic pathways (20). On the other hand, patients who had a positive FIT had more adverse factors that impede the recovery of AIS symptoms, such as electrolyte disorders, paralysis, and alcoholism (21). Finally, FIT-positive patients accompanied by significant evidence of GIB may discontinue anticoagulants or antithrombotic medications, which potentially affect the patient’s prognosis.

The study had limitations. First, this is a retrospective study that cannot draw causal conclusions. Second, the study was single center and included a relatively small number of patients. Third, due to the limitations of the testing method, such as low sensitivity, the results may sometimes be misleading. Large-scale prospective population studies are needed to address limitations by enrolling a larger and more diverse patient population, standardizing data collection across centers, and including additional variables that could influence FIT results and stroke outcomes.

## 5 Conclusion

In conclusion, we demonstrate the relevance of FIT positivity in predicting the increased association of poor neurological outcomes, and high inflammation levels can increase the incidence of positive FIT.

## Data Availability

The raw data supporting the conclusions of this article will be made available by the authors, without undue reservation.
